# Supplementary figures and images for: Computational modeling of color perception with biologically plausible spiking neural networks
Source: PLoS Comput Biol. 2022 Oct 27;18(10):e1010648. doi: 10.1371/journal.pcbi.1010648 (PMC9642903; doi:10.1371/journal.pcbi.1010648)

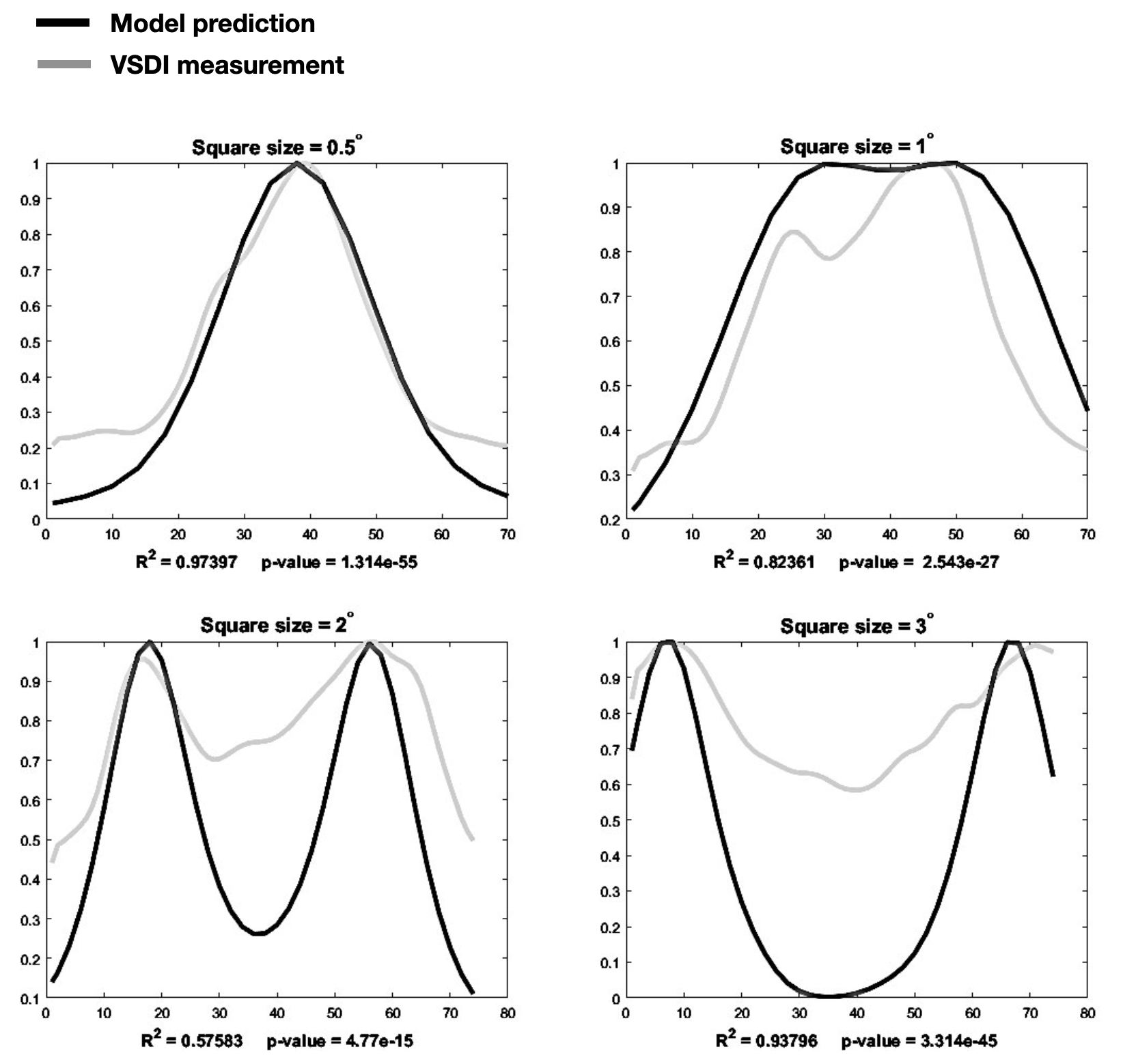

Supplement: S1 Fig — (TIFF) [file pcbi.1010648.s001.tiff]

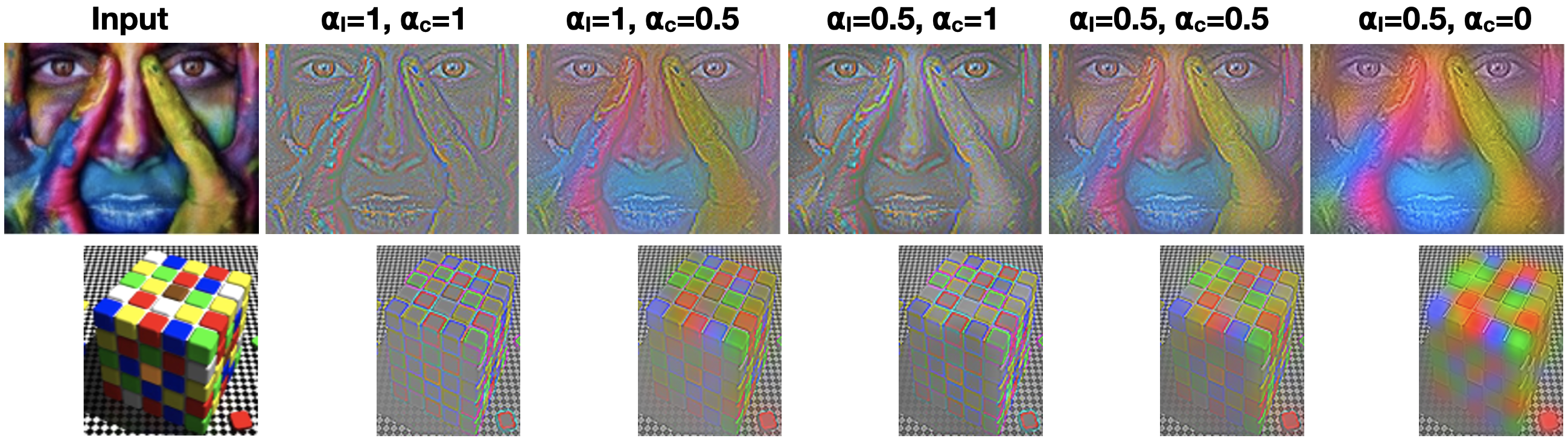

Supplement: S2 Fig — (TIFF) [file pcbi.1010648.s002.tiff]

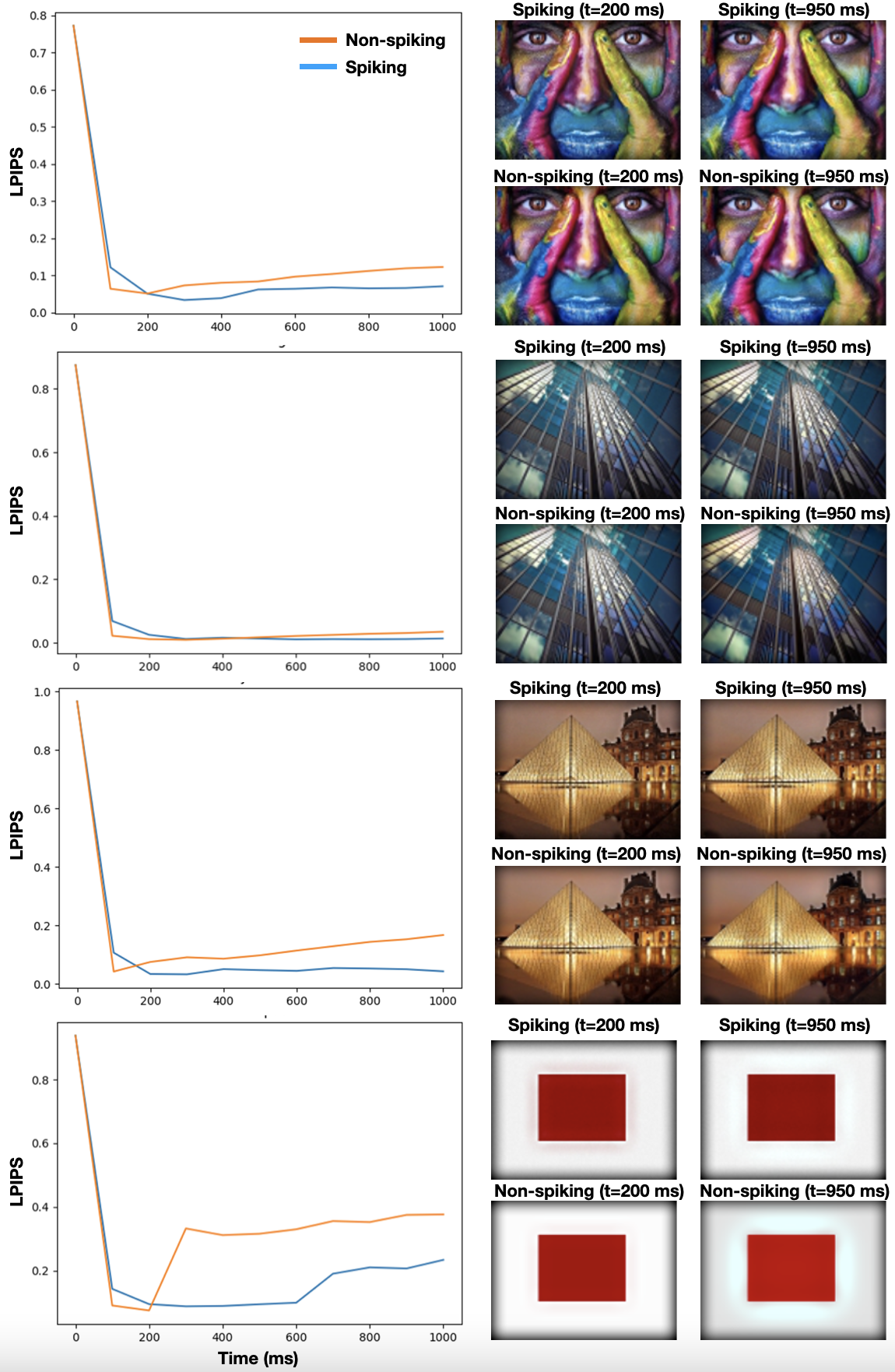

Supplement: S3 Fig — (TIFF) [file pcbi.1010648.s003.tiff]

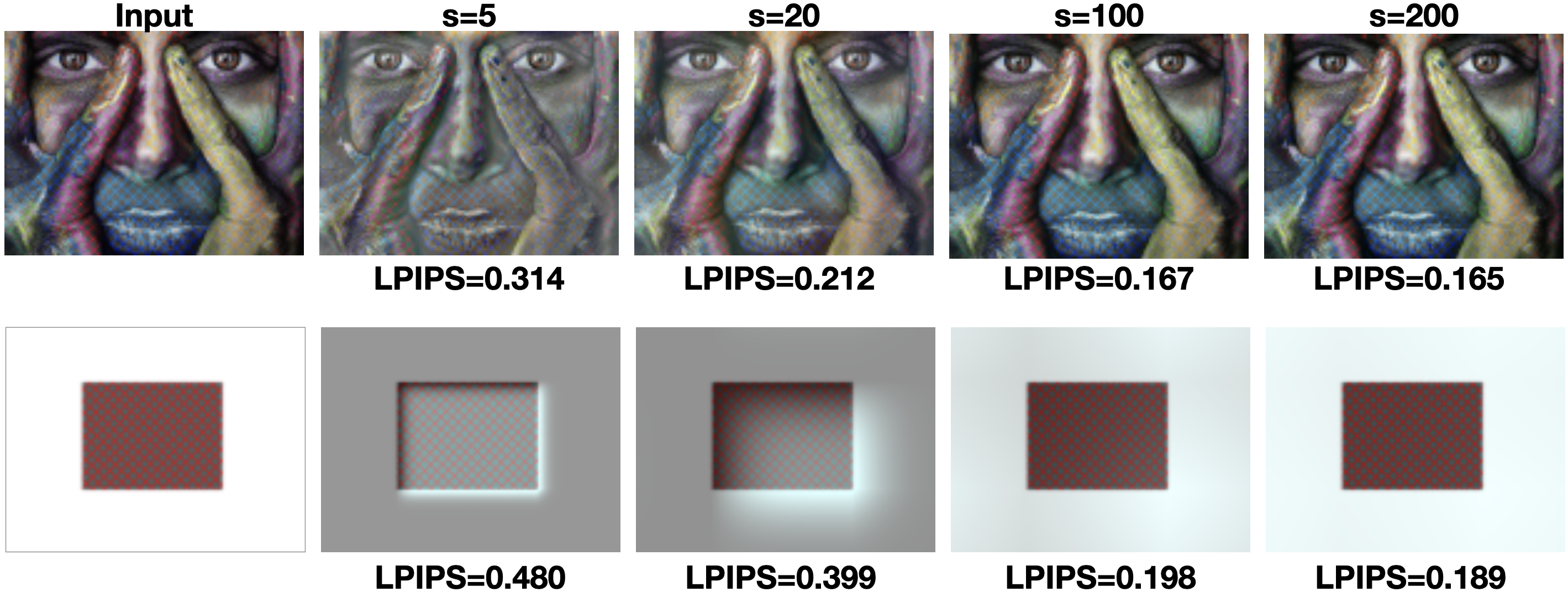

Supplement: S4 Fig — (TIFF) [file pcbi.1010648.s004.tiff]
