# Supplementary material for: Computational modeling of color perception with biologically plausible spiking neural networks
Source: PLoS Comput Biol. 2022 Oct 27;18(10):e1010648. doi: 10.1371/journal.pcbi.1010648 (PMC9642903; doi:10.1371/journal.pcbi.1010648)
Supplement: Attachment — Submitted filename: ReviewComments.pdf [file pcbi.1010648.s005.pdf]

Cohen-Duwek and colleagues implemented a modified SNN-driven model, with low and high spatial frequency filters for both chromatic and achromatic visual information, to explain both VSD imaging data measured in monkey V1 and the perception for color constancy, color assimilation and ambiguous colors. They demonstrated that such a single computational framework provides adequate color perception; and interestingly parameter changes in the model also explain individual differences for perceiving colors. Overall, the evaluation of critical model parameters for color perception, which suggested important roles for both single and double opponent channels, is very interesting; and the demonstration of how a SNN model combined with Poisson-processes to explain visually-driven responses in space and time is also important for further computational and perceptual studies. However, I do have comments and suggestions for revising the manuscript.

1) Model descriptions require more details and explanation. It is quite hard to follow the model without reading several times. I understand that the model is a modified one, which was built for accounting for neural responses driven by achromatic stimuli; but this doesn't mean that some details of the model should be just referred to other paper. A), in the part for NEF, what type of computer language was used? The left side of the equation 1 is a function of x, but on the right side of the equation, there was no explicit x. Is the $a_i(x)$ in equation 2 the same as a_i in equation 1? B) In the description of single and double opponent channels, spatial extent (sigma) of single opponent Gaussian spatial kernel was set at 5 pixels, but the operational matrix for double opponent channel (Laplacian operation) suggest its spatial extent is 1 pixel. The authors should give some explanations for why choosing spatial extents in this way. C), in the part of perceptual filling-in with spiking neurons, the feedback and tau in equation 13 were not explained. In this part, I suggest to change the subscripts for I_s, because it is too similar to I_k or I_{k-1}, which has different meaning. In the last paragraph of this part, 'Therefore, this connectivity scheme can be… as horizontal' is incomplete. Please revise it. D) In the part of image perception, how is the function FI (for delta RG, delta BY and delta I) related to spikes in SNN model? A more explicit equation or explanation should be provided.

2) Results based on model simulation should have more discussions. The study used a model with SNN to explain VSD results. The author should have some discussion on the relationship between VSD signals and spiking activity in V1. The SNN model is a single layer network, which receives retinal input and simulates the neural activity in V1, presumably the superficial layer of V1 (V1 output layer). However, neurons in V1 output layer, in fact, mainly receive excitatory drives from V1 input layer, whose response properties might be different from those in the retina. I am wondering whether using different input for the model can generate different results for parameter alpha or beta. A recent study has demonstrated different spike activity patterns evoked by square stimuli at different V1 layers (Yang et al. 2022, Nature Communications). The author should discuss how their model will perform and whether their conclusion might be held, if the model receives input drive from V1 input layer shown in Yang et al. (2022).

3) Although several color related perceptions have been explored in this study, the

conclusion for each result section should be written more clearly; and their relationship should be discussed. Can the parameter alpha from each section fit to an individual?

4) On page 6, it was written ‘Finally, the resulting surfaces were linearly combined with single opponent outputs to …’. How does this description fit to the later statement, on page 17, ‘rather than combine the ….’. I didn’t quite follow the ‘double-opponent responses as triggers for diffusive Poisson-driven recurrent SNNs’. Please adjust the corresponding method section to make this point more clear.

5) It is interesting to see that different alpha value for chromatic channel will lead to individual difference for perceiving the color of the dress. Gegenfurtner et al. 2015 (Current biology) should be discussed as well.

6) There were two figures for Figure 4 in the main text!
