# Supplementary material for: Computational modeling of color perception with biologically plausible spiking neural networks
Source: PLoS Comput Biol. 2022 Oct 27;18(10):e1010648. doi: 10.1371/journal.pcbi.1010648 (PMC9642903; doi:10.1371/journal.pcbi.1010648)
Supplement: Attachment — Submitted filename: Response Letter.docx [file pcbi.1010648.s006.docx]

**Response Letter**

**Computational modeling of color perception with biologically plausible spiking neural networks**

We would like to thank the reviewers for the time they took to evaluate our work. We were happy to learn that the reviewer found our work interesting and that it might prove useful to the community.
We would like to express our most profound appreciation for their constructive feedback. We did our best to adequately address the reviewers’ comments.

We respectively asked to refer the reviewer to the following substantial revisions we made to the resubmitted manuscript:

1. We significantly extended the result section by adding one chapter discussing parameter evaluation, and a second chapter, discussing model comparison with Retinex, an established model for retinal computing.
2. We’ve added three new figures, two were added as supplementary information and one to the body of the article (figure 7).
3. We extensively revised the discussion to describe our results in the context of the prior art, while addressing the reviewers’ concerns.
4. We revised the mathematical notations and added the suggested extensions.
5. We did our best to improve the language, correct typos, and improve phrasing.
6. Following acceptance, will make our project’s GitHub available to the community, with the entire codebase along with the required documentation. The code base is currently available in: https://github.com/NBELab/PLoS_Comp_2022

We believe that the resubmitted manuscript is significantly strengthened, and we will gladly address any additional revisions and feedback from the reviewers.

**Referee 1**

**We would like to thank the reviewer for the time he/she to evaluate our work. We would like to express our most profound appreciation for his/her constructive feedback. We did our best to adequately address the reviewers’ comments. We believe that the resubmitted manuscript is significantly strengthened, and we will gladly address any additional revisions and feedback from the reviewer.**

**Point-by-Point discussion**

While the proposed model consisted of multiple components, it’s unclear to me whether they are all useful. By assigning different weights to the single-opponent and double-opponent pathways, it could be seen they played different roles in different tasks. However, it’s still unclear whether SNN is necessary. More thorough investigations are needed for the readers to understand the specific contributions made by different components of the model.

**We thank the reviewer, for his/her observation. We extended the discussion to further discuss the biological plausibility of the model, as well as its contribution.**

**Briefly, In the brain, the weighted contribution of the single and double opponent cell channels is determined by visual processes that were not computationally modeled. These parameters might be computed locally within the brain, rather than globally, as in our current framework. When individual perceptual differences are considered (e.g., #theDress and #theShoe) the weighting scheme vary among individuals and can even be different among images within an individual. These parameters can be determined using an unknown set of visual features, supporting the concept of having them derived individually for each image. Therefore, we emphasized in the revised discussion that our model does not suggest the existence of a single set of parameters with which all illusions can be accounted for, nor does it suggest that a single set of parameters would be sufficient for modeling individual color perception. We rather suggest that a weighted combination of the single opponent and the double opponent cells might be sufficient to model individual color perception.**

**To highlight the contribution of our model, we compared it to an established, not-biologically plausible model – the Retinex (Land, 1971) (new Figure 7). Please see further description in our response to the 3^rd^ concern of the reviewer. Retinex's results verify our model predictions regarding the individual perceptual differences as well as the perception of color under different illuminations. In contrast to Retinex, our proposed model is biologically plausible, allowing the attribution of these differences to the proportions between the single and double opponent cells' activity. Furthermore, Retinex was not able to predict as accurately the color assimilation effect (*new* supplementary figure S2), showcasing the generality of our proposed computational framework.**

While the model reproduced three phenomena related to color perception, the exact optimal parameters are different across contexts--the double-opponent component plays an important role in color constancy but not color assimilation. This inconsistency worries me. How does our brain determine which parameter should be used under certain situation? By arbitrarily assigning weights to the two components seems more like explaining different phenomena with different models.

**This concern is directly related to the reviewer’s first concern. We highlighted in the revised discussion that our suggested current model merely demonstrates how different parameters can cause different color perceptions. It does not explain the mechanism that determines these parameters/weights in the brain. To the best of our knowledge, the precise channels' weight setting is still unknown. However, our model adds biological plausibility to the existing state-of-the-art, allowing the attribution of perceptual differences to the proportions between the single and double opponent cells' activity. Furthermore, we show increased generality as our model further accounts for color grid assimilation.**

Most of the analyses in the paper are qualitative in nature, making it difficult for the readers to judge the significance of this paper. In particular, as the authors mentioned, other models have also been proposed to account for color perception. I think it will be much more informative to also examine the performance of other baseline models on the same set of tasks, so we could better appreciate how this new model improves our understanding of color perception.

**We thank the reviewer for his/her important suggestion. We further compared the prediction of the model to a single-scale non-logarithmic version of the Retinex algorithm, one of the most established retinal models in computational vision. Results are shown in Figure 7 (*new* figure). As was mentioned above, Retinex's results verify our model predictions regarding the individual perceptual differences in the images of #theDress and #theShoe as well as the perception of color under different illuminations. In contrast to Retinex, our proposed model is biologically plausible, allowing the attribution of these differences to the proportions between the single and double opponent cells' activity. Furthermore, in contrast to our model, Retinex was not able to predict as accurately the color assimilation effect (*new* supplementary figure S2), showcasing the generality of our proposed computational framework.**

The definition of the coefficients (α and β) in equation 17 seems to be inconsistent with elsewhere in the paper, e.g. Figure 1.

**Corrected in the revised manuscript**

There are two Figure 4 in the figure legends.

**Corrected in the revised manuscript**

**Referee 2**

**We would like to thank the reviewer for the time he/she to evaluate our work. We would like to express our most profound appreciation for his/her constructive feedback. We did our best to adequately address the reviewers’ comments. We believe that the resubmitted manuscript is significantly strengthened, and we will gladly address any additional revisions and feedback from the reviewer.**

**Point-by-Point discussion**

Comparison to V1 data. In Figure 1 the authors show the topographic map of neural activity in monkey V1 to black and red squares. The authors claim (without any supporting statistical tests) that simulation results correspond to the observed neuronal activations in V1.

**We thank the reviewer for his/her important comment. We further compared the reconstructed VSDI profiles with the recorded VSDI profiles using linear regression. Results were added to the results section. The stimulus edges in each VSDI profile were aligned (across the x-axis of each profile) to its corresponding simulated profile and** $\boldsymbol{R}^{\boldsymbol{2}}$ **and its corresponding p-value was derived (*new* supplementary figure S1). Results shows** $\boldsymbol{R}^{\boldsymbol{2}}$ **of** $\boldsymbol{0.974}$**,** $\boldsymbol{0.824}$**,** $\boldsymbol{0.575}$**,** $\boldsymbol{0.938}$ **and p-values of** $\boldsymbol{1.3\cdot}\boldsymbol{10}^{\boldsymbol{-55}}$**,** $\boldsymbol{2.5\cdot}\boldsymbol{10}^{\boldsymbol{-27}}$**,** $\boldsymbol{4.7\cdot}\boldsymbol{10}^{\boldsymbol{-14}}$**,** $\boldsymbol{3.3\cdot}\boldsymbol{10}^{\boldsymbol{-45}}$ **for the 0.5°, 1°, 2° and 3° profiles, indicating a good model fit.** $\boldsymbol{R}^{\boldsymbol{2}}$ **was not calculated in the 8° profile since the signal comprised mostly noise.**

How did the authors choose their parameters (alpha_i and alpha_c) decided? What happens for other beta and alpha values? The authors should show us the results for these choices that change over the course of the paper.

**Briefly, in our suggested model,** $\boldsymbol{\alpha}_{\boldsymbol{i}}$ **and** $\boldsymbol{\alpha}_{\boldsymbol{c}}$ **are two parameters that can be modified to describe various visual phenomena. To demonstrate how the model can generate different perceptions, we modulated these parameters. As the reviewer mentioned, when individual perceptual differences are considered (e.g., #theDress and #theShoe),** $\boldsymbol{\alpha}_{\boldsymbol{i}}$ **and** $\boldsymbol{\alpha}_{\boldsymbol{c}}$ **are vary among individuals and can even be different among images within an individual. As a result, different images might be generated by a different weighting scheme. These parameters can be determined using an unknown set of visual features, supporting the concept of having them derived individually for each image. Therefore, we emphasized in the revised discussion that our model does not suggest the existence of a single set of parameters with which all illusions can be accounted for, nor does it suggest that a single set of parameters would be sufficient for modeling individual color perception. We rather suggest that a weighted combination of the single opponent and the double opponent cells might model individual color perception.**

**To address the reviewer’s concern, in the revised work we further demonstrate the effect** $\boldsymbol{\alpha}_{\boldsymbol{i}}$ **and** $\boldsymbol{\alpha}_{\boldsymbol{c}}$ **have on our model, simulations were conducted with different** $\boldsymbol{SO's}$**spatial parameters (**$\boldsymbol{W}$ **and** $\boldsymbol{\sigma}$**),** $\boldsymbol{\alpha}_{\boldsymbol{i}}$ **and** $\boldsymbol{\alpha}_{\boldsymbol{c}}$**. The simulation results over a CIELu'v' color space for both** $\boldsymbol{\alpha}_{\boldsymbol{i}}$ **and** $\boldsymbol{\alpha}_{\boldsymbol{c}}$ **(ranging from 0.5 to 1) are shown in the *new*** **Figure 7. With each image (colored face, cube with natural-yellowish-blueish illumination, #theDress, and #theShoe), we sampled pixels from different locations in the image where each location has a different color (hue) and ran simulations with varying** $\boldsymbol{\alpha}_{\boldsymbol{i}}$ **and** $\boldsymbol{\alpha}_{\boldsymbol{c}}$**. For further parameter evaluation, we also changed the kernel size of the** $\boldsymbol{SO}$ **cell (**$\boldsymbol{W}$ **= 21,** $\boldsymbol{\sigma}$ **= 5,** $\boldsymbol{W}$ **=11 and** $\boldsymbol{\sigma}$ **= 3; Equations 4-6). It appears that predictions can range over areas of CIELu'v' color space as well as curves. As well, the model appears to be more sensitive to the selection** $\boldsymbol{\alpha}_{\boldsymbol{i}}$ **and** $\boldsymbol{\alpha}_{\boldsymbol{c}}$ **rather than it is to the selection of the spatial parameters of SO cells. These results are now described in the new chapter: parameter evaluation.**

Why do the authors smooth the results instead of just showing us the average responses for all the model units (as in the original paper).

**Since VDSI responses can become noisy, particularly when activity is low, they are often smoothed along the x-axis, allowing the deviation of the activity pattern – the main concerning point here.**

Do the authors feel that these data provide strong constraints on their specific model?

**The purpose of these comparisons was to support our dynamic recurrent model that predicts an isomorphic spread of neural activities from edges to the center of the reconstructed shape. The filling-in strength is determined by the size of the square, where small squares are filled-in while larger squares are not. We believe that the similar filling-in profiles retrieved across both simulations and VSDI support our SNN-driven recurrent filling-in model.**

The authors should do more to quantify the match to data using appropriate statistical tests. They should also do some sensitivity analyses to show the dependence of their claim on the parameters of their model.

**As was described above, in the revised work we further compared the reconstructed VSDI profiles with the recorded VSDI profiles using linear regression and demonstrated the effect** $\boldsymbol{\alpha}_{\boldsymbol{i}}$ **and** $\boldsymbol{\alpha}_{\boldsymbol{c}}$ **have on our model, simulations were conducted with different** $\boldsymbol{SO's}$**spatial parameters (**$\boldsymbol{W}$ **and** $\boldsymbol{\sigma}$**),** $\boldsymbol{\alpha}_{\boldsymbol{i}}$ **and** $\boldsymbol{\alpha}_{\boldsymbol{c}}$**.**

Was the goal of this simulation to find the model parameters that would give the lowers overall LPIPS score?

**We agree with the reviewer that this point should be better clarified in the text. We extended the discussion accordingly.**

**Briefly,** **LPIPS is a measure of perceptual similarity that has been compared to the perceptual results of human subjects. A modification to an image can be compared to a GT target image, based on the LPIPS measurement. The image with the lowest LPIPS score is the one that is most perceptually similar to the GT. While LPIPS can be used to compare two images, it can't account for the differences between an image and its corresponding perception in the brain. Therefore, in our work, LPIPS scores were used not to identify which parameters gave the lowest scores, but rather to illustrate: 1) the model can reconstruct images that are perceptually similar to GT; 2) that the demonstrated illusions which are perceived by the brain are not similar to the GT, resulting in a higher LPIPS score; and 3) that color perception varies across individuals. If someone perceives #theDress as black and blue, then their perception is more similar to the original/physical colors of the GT.**

Given the model is image computable, why do the authors then not just repeat the simulations on many stimuli (say at least 100 or so)? That way we can be sure about the the result here which is that 0.5 is the current model parameter for a good image reconstruction. There is no motivation provided for using these 4 stimuli at all. I am sure the readers would be left wondering about the same question as well… If the best model parameters are 0.5 for image reconstruction, how do the authors justify their original choice for Part 1 of the paper?... The authors next go after, what I feel is the core problem, of color perception – color constancy. The claim here is that the model can factor out the effects of overall illumination and retrieve the true color of the rubiks cube – the model parameters for the best possible reconstruction is now a different number than used for the previous 2 sections… The color assimilation grid illusion. Here too the parameters change to a completely different number

**We agree with the reviewer that this point should be better clarified in the text. We extended the discussion accordingly.**

**As discussed above, each image is perceived differently depending on its context, and this may vary between individuals. different images might be generated by a different weighting scheme. Therefore, we emphasized in the revised discussion that our model does not suggest the existence of a single set of parameters with which all illusions can be accounted for (e.g., having 0.5 being the “right” coefficient), nor does it suggest that a single set of parameters would be sufficient for modeling individual color perception. We rather suggest that a weighted combination of the single opponent and the double opponent cells might model individual color perception. We, therefore, chose 4 stimuli, which in our opinion represent a well know and easily related visual scenes (face, shapes, environment, etc.).**

Overall issue about the parameters. The authors need to do more to convince the readers that the model parameter choice is not just double-dipping into the results. I can think of a few ways of doing this but let me suggest one.

**To address the reviewer’s concern, in the revised work we further demonstrate the effect** $\boldsymbol{\alpha}_{\boldsymbol{i}}$ **and** $\boldsymbol{\alpha}_{\boldsymbol{c}}$ **have on our model, simulations were conducted with different** $\boldsymbol{SO's}$**spatial parameters (**$\boldsymbol{W}$ **and** $\boldsymbol{\sigma}$**),** $\boldsymbol{\alpha}_{\boldsymbol{i}}$ **and** $\boldsymbol{\alpha}_{\boldsymbol{c}}$**. The simulation results over a CIELu'v' color space for both** $\boldsymbol{\alpha}_{\boldsymbol{i}}$ **and** $\boldsymbol{\alpha}_{\boldsymbol{c}}$ **(ranging from 0.5 to 1) are shown in the *new*** **Figure 7.**

**Moreover, we further compared the prediction of the model to a single-scale non-logarithmic version of the Retinex algorithm, one of the most established retinal models in computational vision. Results are shown in Figure 7 (*new* figure). The Retinex's results verify our model predictions regarding the individual perceptual differences in the images of #theDress and #theShoe as well as the perception of color under different illuminations. In contrast to Retinex, our proposed model is biologically plausible, allowing the attribution of these differences to the proportions between the single and double opponent cells' activity. Furthermore, in contrast to our model, Retinex was not able to predict as accurately the color assimilation effect (*new* supplementary figure S2), showcasing the generality of our proposed computational framework.**

For example, the authors could set the parameters based on the neural data (part 1 of the paper) and show the reconstruction results for the same parameters for the other experiments. This is the strongest way to do it – you are defining the internals of the model based on some (albeit weak) neural constraint and then testing reconstruction (perception/behavior) based on those parameters. I am afraid that the current claims would not hold if the authors, or would at least need to be tempered, if the authors choose to do this.

**We agree that this point should be clarified in the text. Hopefully, now, the revised discussion makes our choice of parameters clearer. The revised manuscript highlights that we do not argue for the existence of a single set of parameters would fit all illusions, nor did we suggest that a single set of parameters would be fixed to model individual color perception. The weights/parameters seem to be dependent on the context of the image and vary among individuals. We merely suggested that a weighted combination of the single opponent and the double opponent cells might determine color perception while being general enough to account for myriad visual phenomena.**

Introduction -- Color constancy is not an illusion

**Corrected in the revised manuscript**

Typo – “as well as filtering” under the Color constancy section

**Corrected in the revised manuscript**

**Referee 3**

**We would like to thank the reviewer for the time he/she to evaluate our work. We would like to express our most profound appreciation for his/her constructive feedback. We did our best to adequately address the reviewers’ comments. We believe that the resubmitted manuscript is significantly strengthened, and we will gladly address any additional revisions and feedback from the reviewer.**

**Point-by-Point discussion**

Model descriptions require more details and explanation. It is quite hard to follow the model without reading several times. I understand that the model is a modified one, which was built for accounting for neural responses driven by achromatic stimuli; but this doesn’t mean that some details of the model should be just referred to other paper.

**We agree with the reviewer and appreciate his/her advice. We extended our model description accordingly in the revised manuscript. Furthermore, along with the paper’s publication, we will make our project’s Github public, making our codebase and all implementation details available. The code base is currently available in: https://github.com/NBELab/PLoS_Comp_2022**

In the part for NEF, what type of computer language was used? The left side of the equation 1 is a function of x, but on the right side of the equation, there was no explicit x. Is the ai(x) in equation 2 the same as ai in equation 1?

**The reviewer is correct, equation 2 has been corrected accordingly. We used Nengo, a Python library that provides a full implementation of NEF. We’ve added this information to the text and provided the relevant reference for the library description.**

In the description of single and double opponent channels, the spatial extent (sigma) of single opponent Gaussian spatial kernel was set at 5 pixels, but the operational matrix for double opponent channel (Laplacian operation) suggest its spatial extent is 1 pixel. The authors should give some explanations for why choosing spatial extents in this way.

**We agree with the reviewer that this part should be better clarified in the text. For the** $\boldsymbol{DO}$ **cells, the spatial extents of the filters were used to represent a high-pass filter (Laplacian), and for the** $\boldsymbol{SO}$ **cells, we chose a low-pass filter with a relatively large support (wide spatial Gaussian profile).**

In the part of perceptual filling-in with spiking neurons, the feedback and tau in equation 13 were not explained. In this part, I suggest to change the subscripts for Is, because it is too similar to Ik or Ik-1, which has different meaning. In the last paragraph of this part, ‘Therefore, this connectivity scheme can be… as horizontal’ is incomplete. Please revise it.

**We agree with the reviewer that this part should be better clarified in the text. In all simulations, we used a tau of 0.25. We clarified it in the “Model simulation” section. We also changed the subscription and rephrased the final paragraph as suggested by the reviewer.**

In the part of image perception, how is the function FI (for delta RG, delta BY and delta I) related to spikes in SNN model? A more explicit equation or explanation should be provided.

**We added a description and the additional equations (under the image perception section) as suggested by the reviewer (new Equations. 17-19).**

Results based on model simulation should have more discussions. The study used a model with SNN to explain VSD results. The author should have some discussion on the relationship between VSD signals and spiking activity in V1. The SNN model is a single layer network, which receives retinal input and simulates the neural activity in V1, presumably the superficial layer of V1 (V1 output layer).

**We thank the reviewer for raising this point, which we clarified in the revised manuscript. The model simulates the output layers of V1 to other cortical areas, i.e. layers 2-3 which are also the upper layers. Layers 2-3 are also the main cortical layers imaged by VSDI, a technique that measures the membrane potential from a population of neurons (rather than the response of single neurons) at high spatial resolution (mesoscale, 502 μm2/pixel) and temporal resolution (100 Hz) simultaneously (Shoham et al., 1999; Grinvald and Hildesheim, 2004; Slovin et al. 2002). The VSD signal emphasizes subthreshold membrane potentials but reflects also supra-threshold membrane potentials (i.e., spiking activity; see Ayzenshtat et al. 2010; Grinvald and Hildesheim 2004; Jancke et al. 2004). The main advantage of this technique is the combination of wide-field imaging with high spatio-temporal resolution, which enables the visualization of complete cortical patterns evoked by a visual stimulus.**

Neurons in V1 output layer, in fact, mainly receive excitatory drives from V1 input layer, whose response properties might be different from those in the retina. I am wondering whether using different input for the model can generate different results for parameter alpha or beta.

**In this work, we simulated the single opponent and double opponent neurons in V1 after receiving the retinal input. A clarification was added to the text.**

A recent study has demonstrated different spike activity patterns evoked by square stimuli at different V1 layers (Yang et al. 2022, Nature Communications). The author should discuss how their model will perform and whether their conclusion might be held, if the model receives input drive from V1 input layer shown in Yang et al. (2022).

**We thank the reviewer for his reference, which we now refer to in the revised manuscript. The results in Yang et al. 2022 paper are very similar to the VSD results that we report here. In particular, Yang et al. showed that V1 responses in layers 2-3 are enhanced at the surface edges whereas the response at the surface center is suppressed. This is highly similar to the results reported here and in previous VSDI studies on V1 encoding of luminance surfaces (Zweig et al. 2015; Zurawel et al. 2014). However, while Yang et al. used a square surface of one size (4X4 deg), in our work we used a range of surfaces size. Using a range of surfaces sizes and computing the slope of population propagation from the edges to the center of the surface, we found in previous studies (Zweig et al. 2015; Zurawel et al. 2014) that horizontal connections contribute to the filling-in phenomenon at the surface center, however, this contribution is dependent on the surface size. We revised the discussion, addressing this point.**

Although several color related perceptions have been explored in this study, the conclusion for each result section should be written more clearly; and their relationship should be discussed. Can the parameter alpha from each section fit to an individual?

**We thank the reviewer for this comment. As was described in length above in our response to the 1^st^ and 2^nd^ reviewers, in the revised work we further demonstrate the effect** $\boldsymbol{\alpha}_{\boldsymbol{i}}$ **and** $\boldsymbol{\alpha}_{\boldsymbol{c}}$ **have on our model, simulations were conducted with different** $\boldsymbol{SO's}$**spatial parameters (**$\boldsymbol{W}$ **and** $\boldsymbol{\sigma}$**),** $\boldsymbol{\alpha}_{\boldsymbol{i}}$ **and** $\boldsymbol{\alpha}_{\boldsymbol{c}}$**. The simulation results over a CIELu'v' color space for both** $\boldsymbol{\alpha}_{\boldsymbol{i}}$ **and** $\boldsymbol{\alpha}_{\boldsymbol{c}}$ **(ranging from 0.5 to 1) are shown in the *new* Figure 7. Moreover, we further compared the prediction of the model to a single-scale non-logarithmic version of the Retinex algorithm, one of the most established retinal models in computational vision. Results are shown in Figure 7 (*new* figure).**

On page 6, it was written ‘Finally, the resulting surfaces were linearly combined with single opponent outputs to …’. How does this description fit to the later statement, on page 17, ‘rather than combine the ….’. I didn’t quite follow the ‘double-opponent responses as triggers for diffusive Poisson-driven recurrent SNNs’. Please adjust the corresponding method section to make this point more clear.

**We amended the methods section to better clarify this point. In a diffusion-like process, the double opponent cell activates its neighbors. The recursive equation - Eq. 16 - expresses this process.**

It is interesting to see that different alpha value for chromatic channel will lead to individual difference for perceiving the color of the dress. Gegenfurtner et al. 2015 (Current biology) should be discussed as well.

**We revised the discussion to address the reviewer's accurate suggestion. As was demonstrated by the model's prediction of #TheDress and #TheShoe, despite having only global parameters, our model was able to capture differences in perception with respect to the model’s parameters (*new* Figure 7). Thus, concurring with Gegenfurtner and colleagues, who found that there are multiple answers to the question, “what color is the dress?” [63]. Our results extend their finding and demonstrate in a biologically plausible computational framework that there can be multiple responses to any color image. In the colored face image, for example, people may perceive and name colors differently. Some may name the orange part of the woman's finger orange, while others might call it yellowish or greenish (since the original orange color changed to greenish-yellowish, as the alpha changed (Figure 7, top row).**

There were two figures for Figure 4 in the main text

**Corrected in the revised manuscript**
