# Supplementary material for: Computational modeling of color perception with biologically plausible spiking neural networks
Source: PLoS Comput Biol. 2022 Oct 27;18(10):e1010648. doi: 10.1371/journal.pcbi.1010648 (PMC9642903; doi:10.1371/journal.pcbi.1010648)
Supplement: Attachment — Submitted filename: Response Letter R2.docx [file pcbi.1010648.s007.docx]

**Response Letter**

**Computational modeling of color perception with biologically plausible spiking neural networks**

We would like to thank the 2^nd^ and 3^rd^ reviewers for their endorsement for publication.

We would like to thank reviewer 1, for providing us with further guidelines, allowing us to improve the presentation of our work.

We respectively asked to refer the reviewer to the following substantial revisions we made to the resubmitted manuscript:

1. We’ve added two new supplementary figures (S2, S3), addressing the reviewer’s concerns.
2. We extensively revised the discussion to describe our results in the context of the prior art, while addressing the reviewer’s concerns.

We believe that the resubmitted manuscript is significantly strengthened, and we will gladly address any additional revisions and feedback from the reviewer.

**Referee 1**

**We would like to thank the reviewer for the time he/she to evaluate our work. We would like to express our most profound appreciation for his/her constructive feedback. We did our best to adequately address the reviewers’ comments. We believe that the resubmitted manuscript is significantly strengthened, and we will gladly address any additional revisions and feedback from the reviewer.**

**Point-by-Point discussion**

What happens when SNN is absent from the model, such as when the SO and DO channels are combined directly, or when a regular neural network is employed in its place?

**There are two parts to the question:**

**1) What happens when SO and DO are connected without a filling-in network.**

**2) What happens when a non-spiking network is employed.**

**Point 1.** **Following the reviewer’s concern,** **we evaluated the importance of the filling-in component by removing it from the proposed perceptual pipeline. Without a recurrent connection between the SO and DO channels, our model is simplified to a combination of low- and high-pass filters where most of the band-pass signals (intermediate frequencies) are absent. When** $\boldsymbol{\alpha}_{\boldsymbol{i}}$ **and** $\boldsymbol{\alpha}_{\boldsymbol{c}}$ **equal 1, the resulted reconstruction is a high-pass filter of the image (the image's Laplacian), whereas decreasing alphas merely adds low frequencies to the results. We discussed the results in the revised text and added a Supplementary Figure S2 to the revised submission to support it.**

**Point 2. Following the reviewer’s concern, we further evaluated the proposed model with a non-spiking version (a conventional neural network). A SNN is considered biologically plausible is it uses spikes to represent and transform data through local learning rules (Equation 2). However, we can analytically solve the mathematical transformation our SNN strives to approximate (same network architecture, no spike-based neural approximation). Under visual inspection, the reconstructed results of the spiking and non-spiking neural networks are similar, pointing out the capacity of our model to exhibit relevant neural approximations. Interestingly, when measuring LPIPS distances during models’ convergence, the SNN outperform the conventional neural network, consistently reporting lower distances (Supplementary Figure S3). This might be due to the noise-introductory effect, which is inherit in SNNs being a neural approximation, to the resolved diffusion process (Equation 16). While here we used a SNN to increase the biological plausibility of the model, the improved diffusive filling-in process may be an interesting topic to investigate in a future work. We discussed the results in the revised text and added a Supplementary Figure S3 to the revised submission to support it.**

What biological process controls how much weight is given to SO and DO channels? I think there should be some explanation for these weightings because they should be the outcome of biological processes, especially given the authors' emphasis on biological plausibility.

**To the best of our knowledge, the underlying mechanism for SO/DO weighting scheme is largely unknown. However, we understand the reviewer’s concern. We therefore significantly extended the discussion, succinctly discussing recent approaches, while more accurately pin-pointing the contribution of our model to this important discussion.**
